# Exposure to violence and mental health of adolescents: South African Health and Well-being Study

**DOI:** 10.1192/bjpo.bp.117.004861

**Published:** 2017-10-17

**Authors:** Stephen A. Stansfeld, Catherine Rothon, Jayati Das-Munshi, Cathy Mathews, Arlene Adams, Charlotte Clark, Crick Lund

**Affiliations:** **Stephen A. Stansfeld**, PhD FRCPsych, Centre for Psychiatry, Wolfson Institute of Preventive Medicine, Barts and the London, School of Medicine and Dentistry, Queen Mary University of London, London, UK; **Catherine Rothon**, PhD, St George’s University of London, London, UK; **Jayati Das-Munshi**, PhD MRCPsych, Department of Health Service and Population Research, Institute of Psychiatry, Psychology and Neuroscience (IoPPN), Kings College London, London, UK; **Cathy Mathews**, PhD, Health Systems Research Unit, Medical Research Council, Cape Town, South Africa; School of Public Health and Family Medicine, University of Cape Town, Cape Town, South Africa; **Arlene Adams**, PhD, Department of Student Affairs, University of Cape Town, Cape Town, South Africa; **Charlotte Clark**, PhD, Centre for Psychiatry, Wolfson Institute of Preventive Medicine, Barts and the London, School of Medicine and Dentistry, Queen Mary University of London, London, UK; **Crick Lund**, PhD, Alan J. Flisher Centre for Public Mental Health, Department of Psychiatry and Mental Health, University of Cape Town, Cape Town, South Africa; Centre for Global Mental Health, Institute of Psychiatry, Psychology and Neuroscience, King’s College London, London, UK

## Abstract

**Background:**

Material and social environmental stressors affect mental health in adolescence. Protective factors such as social support from family and friends may help to buffer the effects of adversity.

**Aims:**

The association of violence exposure and emotional disorders was examined in Cape Town adolescents.

**Method:**

A total of 1034 Grade 8 high school students participated from seven government co-educational schools in Cape Town, South Africa. Exposure to violence in the past 12 months and post-traumatic stress disorder (PTSD) symptoms were measured by the Harvard Trauma Questionnaire, depressive and anxiety symptoms by the Short Moods and Feelings Questionnaire and the Self-Rating Anxiety Scale.

**Results:**

Exposure to violence was associated with high scores on depressive (odds ratio (OR)=6.23, 95% CI 4.2–9.2), anxiety (OR=5.40, 95% CI 2.4–12.4) and PTSD symptoms (OR=8.93, 95% CI 2.9–27.2) and increased risk of self-harm (OR=5.72, 95% CI 1.2–25.9) adjusting for gender and social support.

**Conclusions:**

We found that high exposure to violence was associated with high levels of emotional disorders in adolescents that was not buffered by social support. There is an urgent need for interventions to reduce exposure to violence in young people in this setting.

**Declaration of interest:**

None.

**Copyright and usage:**

© The Royal College of Psychiatrists 2017. This is an open access article distributed under the terms of the Creative Commons Non-Commercial, No Derivatives (CC BY-NC-ND) license.

Mental disorders in young people are a worldwide challenge^1^ and there is a need to understand both risk factors for adolescent mental health and potential protective factors.^2^ One important risk factor is exposure to violence which increases the risk of post-traumatic stress disorder (PTSD),^3–5^ depression,^6,7^ suicidal ideation^8^ and risk behaviours.^9^ Exposure to violence may occur in domestic and community settings^5^ and both are related to increased risk of mental ill health. Multiple exposures to adversity will increase risk of mental ill health in adolescence and adulthood.^10^ Adversity, in this context includes exposure to material disadvantage and poverty as well as exposure to lack of care, physical and sexual abuse and violence.

Relatively little is known about the impact of violence on adolescent mental health in South Africa and the possible ameliorating effects of social support especially in inner city communities with high levels of poverty. The degree of entrenched severe structural social inequalities, a legacy of the apartheid era, may uniquely normalise and enhance the use of violence as a method of control in South Africa. Exposure to adversity is strongly linked to socio-economic status in South Africa.^11^ Poverty may influence the risk of mental ill health in several ways, increasing the likelihood of exposure to life events and violence, increasing the likelihood of mental health effects related to violence^12^ and also providing less of a buffer to protect against the consequences of those events. In another paper from this data-set, we have shown that mental ill health is associated with material disadvantage in this sample.^13^ Less advantaged socioeconomic status is also associated with increased risk of revictimisation. In terms of protective processes inducing resilience that may mitigate the effects of stressors, social support has direct protective effects on well-being even after adjusting for neighbourhood and family socioeconomic position and ethnicity.^14^ We hypothesised that (1) exposure to violence would be associated with increased odds of depressive symptoms, anxiety symptoms, symptoms of PTSD and attempted suicide, and (2) that social support would moderate the effects of exposure to violence on the odds of developing mental ill health.

## Method

The sample was drawn from seven government co-educational schools in Cape Town, South Africa. To obtain a representative sample of Cape Town school students, all schools from one administrative district in Cape Town were stratified according to the level of fees charged (high 17 schools, moderate 21 schools and low 23 schools) and two schools chosen randomly from each category. We added up the number of students in each school within each category and selected schools using a random number generator, thereby weighting selection by the number of students in each school. An additional school was added to increase the percentage of White pupils to ensure a sample that was more demographically representative of the Western Cape Province. All Grade 8 pupils in the school were eligible. An equal number of classes were selected in each school regardless of school size. Of 1169 eligible students, 1034 students participated, giving a response rate of 88%. A total of 119 pupils were absent and 16 refused to participate.

Data were collected by self-report questionnaires completed in mixed ability classes. Questionnaires were available in English, Afrikaans and isiXhosa, the three predominant languages spoken in Cape Town. Questionnaires were translated into Afrikaans and isiXhosa and back translated by native speakers. An examination of the internal and retest reliability of the questionnaires was tested in a pilot sample of 237 isiXhosa from the same schools.^15^ Participants were invited to take part in the study and were asked for their written assent. Parents were informed about the study with an information sheet and could opt their children out of the study if they did not wish them to participate. Questionnaires were completed under examination conditions in the classroom. The study was approved by the Western Cape Education Department and the Health Sciences Faculty Human Research Ethics Committee at the University of Cape Town.

Sociodemographic information included age, gender and racially classified social group. Participants were asked to self-identify their ethnicity according to the categories of Black, White, coloured, Indian and ‘other’. These categories were similar to those employed in the South African census. The same categories are employed here although are acknowledged as socially constructed and not as essentialist representations of ‘race’. The use of these categories acknowledges their historical disadvantage.^16^ Household socioeconomic indicators included self-rated financial difficulty, household amenities, grant receipt and parental employment status.

Adversity exposure was assessed by the Harvard Trauma Questionnaire (HTQ), previously used with adolescents in South Africa,^17^ comprising 27 items addressing exposure to violence, including witnessing or being a victim during the past 12 months.^18^ The scores were summed to give a maximum possible score of 27. This scale was abbreviated from a longer version of the scale previously used in Cape Town studies by combining the categories ‘someone I know’ and ‘family member’.^13^ In our pilot study, internal reliability (Cronbach’s alpha 0.818) and test–retest reliability (*r*=0.858) were high.^15^

The HTQ was also used to assess PTSD symptoms. Each question on the scale asked about severity of symptoms on a 4-point Likert scale. Respondents who had an average score of 2 or greater across the questions were deemed to be a case. The 13-item Short Moods and Feelings Questionnaire (SMFQ)^19^ measured core depressive symptoms with a score greater than 7.5 being taken as a possible case indicating significant clinical depressive symptoms. Using this threshold, the SMFQ has been shown to discriminate clinically referred child psychiatric participants from unselected participants with depression in a general population sample.^19^ The 19-item Zung Self-Rating Anxiety Scale (SAS), already used in South African adolescents^15^ measured anxiety symptoms by a threshold of greater than 43 to indicate anxiety ‘cases’, although in other studies this threshold would include ‘subthreshold anxious’.^20^

Social support was measured by the Multi-Dimensional Scale of Perceived Social Support (MSPSS),^21^ a 12-item self-report measure with three response categories assessing social support from: family, friends and significant others. A total score was summed from the family, friends and significant others subscales. It has good concurrent, construct and discriminant validity and high internal and test–retest reliability.^15^

Smoking, drinking and drug use questions were drawn from reliable questions previously used in Cape Town school studies.^22^ Illicit drugs were grouped and questions related to usage in the past month. Other covariates include healthy eating questions originally from the Habit Study; Health Education Authority physical activity questions used in research with the East London Adolescents Community Health Survey.^23^

Parental monitoring was measured by asking respondents five questions about how much their primary caregiver knows about what they do in their spare time and who they spend it with. Possible responses were ‘doesn’t know’ (coded 0), ‘knows a little’ (coded 1) and ‘knows a lot’ (coded 2). These responses were summed to create a scale from 0 to 10 with high scores indicating high levels of parental monitoring.

Based on our previous UK-based study of East London adolescents (RELACHS),^23^ we estimated before recruitment that a sample size of 1150 in the South African Health and Well-being Study (SHaW) would give 99% power (alpha 0.05, two-tailed) to detect a difference in the prevalence of depressive symptoms on the SMFQ of 14% between those with high and low social support.

All data management and analysis was carried out using Stata version 10.0. There were some missing data at baseline: for 49 respondents on depressive symptoms (5%), 85 on anxiety symptoms (8%), 143 respondents on PTSD (14%), 21 respondents on suicidal attempt (2%), 132 respondents on exposure to violence (13%) and 137 respondents on social support (13%). As the amount of missing data was relatively small for most of the key variables, we excluded pupils who did not have complete data.

Because the primary sampling unit for the study was the school, it was necessary to make adjustments for the clustered survey design (using the *svy* commands in Stata). An equal number of classes were selected in each school regardless of school size. Data were, therefore, reweighted to adjust for unequal probabilities of selection.

In univariate analyses, crude odds ratios (ORs) were calculated for the association between each variable and each of the mental health outcomes using logistic regression. We assessed a number of potential confounding factors including gender, ethnicity, religion, social position (self-rated financial difficulty, household amenities, grant receipt, parental employment and mother’s education), general health, health behaviours (physical activity, smoking, alcohol use and drug taking), social support, number of close people and parental monitoring. Confounding factors were assessed by Mantel–Haenszel methods and univariate logistic regression analysis. Confounding factors identified from the univariate analyses were ethnicity, self-rated financial difficulty, household amenities, grant receipt, parental employment, general health, physical activity, smoking, alcohol use, social support and number of close people. Effect modification was investigated by looking at the stratum-specific ORs.

Multivariate logistic regression analysis was carried out adding confounding factors to the model in groups, in the following order: gender, ethnicity, social position variables, general health, health behaviours, social support and number of close people. If adding a confounding factor resulted in an improvement in model fit, they were retained in the model. The Wald test was used to assess model fit. Multiplicative interactions were examined between violence and social support (measured as MSPSS total score, and as a binary variable cut at the median, <67) for each of three mental health outcomes.

We used the Akaike Information Criterion (AIC) to assess goodness of fit of final models. The AIC assesses the fit of models, lower values of AIC suggest a better fit to the data.^24^ For this final assessment of model fit, we declared survey weights and specified the primary sampling unit as a cluster with robust standard errors. The AIC was used to assess the association of increasing quartiles of exposure to violence with mental health outcomes, with violence specified by either a categorical, linear or quadratic term. *P*-values for linear and quadratic trends were derived using Wald tests on survey weighted data.

## Results

Table 1 shows the characteristics of the sample and the odds of exposure to violence. Half of the sample was male, the largest ethnic group was coloured. Mean age was 14.2 years (range 13–19), 95% CI 13.9–14.5. Most reported that they had access to television, electricity and tap water in their homes, although nearly a fifth did not have an indoor bathroom. Most were unable to afford luxury goods, even if they could afford the basic necessities; 33.4% received government grants; two-thirds of mothers and fathers were employed. Many (84.1%) had been exposed to violence. The prevalence of depression was high, with 41.2% cases on the SMFQ; 15.6% with case level anxiety symptoms and 21.5% case level PTSD symptoms; and 13.4% of the sample had attempted suicide.

**Table 1:** Descriptive statistics and odds of exposure to high levels of violence

	% Adjusted for survey design (*N*)	Odds of being in top quartile of exposure to violence (95% CI)
Gender		
Male	46.1 (433)	1
Female	53.9 (507)	0.54 (0.34–0.84)
Ethnicity		
Black	26.5 (276)	1
White	10.0 (102)	0.20 (0.03–1.37)
Coloured	60.6 (603)	0.59 (0.31–1.13)
Indian	2.1 (21)	0.17 (0.04–0.81)
Other	0.8 (8)	0.27 (0.02–4.58)
Physical activity per week		
None	25.1 (255)	1
About half an hour	23.1 (230)	0.99 (0.61–1.60)
About 1 h	19.2 (194)	0.79 (0.39–1.57)
About 2–3 h	17.7 (177)	0.95 (0.36–2.49)
About 4–6 h	8.9 (90)	0.66 (0.09–4.85)
7 h or more	6.0 (61)	0.68 (0.07–6.96)
Smoked a whole cigarette in past week		
No	80.4 (812)	1
Yes	19.6 (197)	3.22 (1.51–6.88)
Used alcohol in past 4 weeks		
No	83.2 (834)	1
Yes	16.8 (170)	4.40 (2.04–9.50)
Smoked marijuana in past 4 weeks		
No	92.0 (930)	1
Yes	8.0 (81)	5.99 (2.83–12.66)
Amenities in home		
Television	97.3 (995)	0.49 (0.06–4.27)
Electricity	98.7 (1012)	0.29 (0.04–1.94)
Tap water	96.4 (988)	0.54 (0.13–2.16)
Motor car or bakkie	68.7 (692)	0.46 (0.18–1.17)
Indoor bathroom	81.6 (827)	0.49 (0.20–1.21)
Type of home		
Shack	7.3 (77)	1
Wendy house (prefabricated shack)	6.3 (66)	1.94 (1.07–3.52)
Brick house	75.5 (761)	0.73 (0.25–2.14)
Flat	9.6 (98)	1.33 (0.78–2.27)
Other	1.2 (12)	0.56 (0.10–2.95)
Self-rated financial difficulty		
Have money for luxury goods and extra things	29.1 (275)	1
Have most important things, few luxury goods	35.2 (333)	1.69 (1.05–2.73)
Enough for food and clothes but short of other things	15.7 (152)	2.44 (0.76–7.79)
Enough for food, not other basic items	12.1 (118)	2.81 (1.48–5.37)
Not enough money for food	7.9 (78)	2.74 (0.98–7.62)
Household receiving grants		
No	66.6 (642)	1
Yes	33.4 (329)	1.68 (0.97–2.91)
Maternal employment		
Yes	67.8 (643)	1
No	25.5 (245)	1.07 (0.47–2.43)
Do not live with mother	6.7 (64)	1.36 (0.58–3.17)
Paternal employment		
Yes	70.1 (666)	1
No	10.7 (104)	1.99 (0.70–5.63)
Do not live with father	19.2 (186)	1.92 (1.22–3.03)
Tertiles of parental monitoring		
Bottom	24.8 (222)	1
Mid	33.8 (303)	0.70 (0.38–1.31)
Top	41.5 (372)	0.42 (0.17–1.05)
Exposed to violence		
Yes	84.1 (869)	
No	15.9 (165)	
Case on SMFQ		
No	58.8 (580)	1
Yes	41.2 (405)	2.89 (1.76–4.77)
Case on SAS		
No	84.4 (801)	1
Yes	15.6 (148)	3.14 (1.66–5.94)
Case on HTQ PTSD scale		
No	78.5 (193)	1
Yes	21.5 (698)	4.58 (2.37–8.85)
Has attempted suicide		
No	86.6 (876)	1
Yes	13.4 (137)	3.62 (1.60–8.18)

SMFQ, Short Moods and Feelings Questionnaire; SAS, Self-Rating Anxiety Scale; HTQ, Harvard Trauma Questionnaire; PTSD, post-traumatic stress disorder.

In univariate analysis of associations with violence, girls and Indian students had lower odds of violence exposure, whereas young people with health risk behaviours, those living in poor housing, with financial difficulties and paternal unemployment and high scorers on the mental health outcomes had higher odds of exposure to violence. Table 2 shows the univariate analyses for the associations between adolescent disorders and demographic risk and protective factors. Depression was more frequent in girls than boys. White and Indian pupils had lower odds of depressive caseness compared with Black pupils, and White pupils and coloured pupils had lower odds of PTSD compared with Black pupils. Being in the highest quartile of exposure to violence was associated with more than five times the odds of depression, anxiety and PTSD caseness than those adolescents in the lowest quartile of exposure to violence. In univariate associations, girls were more likely to have made a suicide attempt than boys (OR=2.18, 95% CI 1.42–3.35). Being in the top quartile of exposure to violence conferred higher odds of attempting suicide compared with the lowest quartile (OR=5.72, 95% CI 1.26–25.95). Drug and alcohol use were also associated with higher odds of attempting suicide. Exposure to violence scored in quartiles was not statistically significantly associated with a dichotomised measure of social support.

**Table 2:** Univariate analysis of factors associated with mental ill health

	Case on SMFQ, OR (95% CI)	Case on SAS, OR (95% CI)	Case on HTQ PTSD scale, OR (95% CI)
Quartile on HTQ			
1	1	1	1
2	1.81 (0.97–3.38)	2.00 (0.97–4.11)	2.44 (0.57–10.40)
3	3.62 (2.35–5.57)	2.09 (0.46–9.47)	3.45 (0.73–16.18)
4	5.49 (2.81–10.71)	5.14 (1.75–15.09)	9.76 (3.14–30.24)
Gender			
Male	1	1	1
Female	1.67 (1.38–2.02)	2.06 (0.93–4.56)	1.19 (0.97–1.46)
Ethnicity			
Black	1	1	1
White	0.38 (0.17–0.88)	0.38 (0.12–1.19)	0.31 (0.21–0.44)
Coloured	0.73 (0.50–1.06)	0.63 (0.36–1.11)	0.46 (0.31–0.67)
Indian	0.34 (0.25–0.47)	-	-
Other	0.75 (0.09–6.50)	-	-
Physical activity/week			
None	1	1	1
About half an hour	0.64 (0.40–1.03)	0.93 (0.52–1.66)	1.03 (0.67–1.60)
About 1 h	0.65 (0.47–0.91)	0.70 (0.49–1.01)	1.13 (0.73–1.76)
About 2–3 h	0.49 (0.34–0.69)	0.82 (0.50–1.33)	0.96 (0.49–1.88)
About 4–6 h	0.42 (0.18–0.94)	0.42 (0.07–2.68)	0.63 (0.10–3.80)
7 h or more	0.52 (0.15–1.73)	1.17 (0.35–3.94)	1.01 (0.28–3.66)
Smoked in past 4 weeks			
No	1	1	1
Yes	2.30 (1.24–4.28)	1.51 (0.79–2.89)	1.81 (0.96–3.38)
Alcohol in past 4 weeks			
No	1	1	1
Yes	2.65 (2.07–3.40)	1.41 (1.00–1.97)	2.12 (1.53–2.93)
Marijuana in past 4 weeks			
No	1	1	1
Yes	2.17 (0.81–5.80)	1.11 (0.42–2.93)	1.99 (0.62–6.37)
Self-rated financial difficulty			
Richest	1	1	1
2	1.78 (0.71–4.44)	1.24 (0.58–2.62)	1.54 (0.74–3.23)
3	2.37 (1.00–5.59)	1.81 (0.63–5.20)	1.75 (0.80–3.82)
4	1.73 (0.83–3.60)	2.10 (0.66–6.70)	2.64 (1.10–6.34)
Poorest	4.70 (2.13–10.39)	3.82 (1.63–8.94)	2.31 (0.72–7.33)
Household amenities			
Less than 4	1	1	1
4 or 5	0.68 (0.40–1.14)	0.56 (0.15–2.12)	0.48 (0.33–0.68)
Household receiving grants			
No	1	1	1
Yes	1.55 (1.01–2.37)	1.62 (0.98–2.70)	1.25 (0.76–2.07)
Maternal employment			
Yes	1	1	1
No	1.05 (0.65–1.69)	1.51 (1.15–1.99)	1.03 (0.65–1.66)
Do not live with mother	2.07 (0.67–6.43)	1.98 (0.90–4.38)	1.87 (1.15–3.03)
Paternal employment			
Yes	1	1	1
No	1.47 (0.65–3.32)	2.14 (0.97–4.70)	2.34 (1.17–4.69)
Do not live with father	1.76 (0.62–5.00)	1.25 (0.68–2.30)	1.46 (0.63–3.36)
Social support total score	0.97 (0.96–0.98)	0.98 (0.97–0.99)	0.99 (0.97–1.01)
Social support friends	0.95 (0.92–0.98)	0.94 (0.92–0.97)	0.98 (0.94–1.02)
Social support family	0.90 (0.86–0.94)	0.92 (0.89–0.96)	0.94 (0.90–0.99)
Social support significant other	0.95 (0.92–0.98)	0.96 (0.92–1.01)	0.99 (0.95–1.03)
Close people (continuous)	0.88 (0.79–0.99)	0.86 (0.70–1.07)	0.96 (0.83–1.12)
Close friends (continuous)	0.91 (0.78–1.05)	0.87 (0.78–0.97)	0.94 (0.76–1.16)
Parental monitoring (continuous)	0.85 (0.78–0.93)	0.94 (0.83–1.08)	0.93 (0.82–1.05)

SMFQ, Short Moods and Feelings Questionnaire; SAS, Self-Rating Anxiety Scale; HTQ, Harvard Trauma Questionnaire; PTSD, post-traumatic stress disorder.

Those in the ‘poorest’ category had nearly five times the odds of depressive caseness and nearly four times the odds of caseness of anxiety compared with those in the ‘richest’ category (Table 2). Reporting a higher number of household amenities was associated with half the odds of PTSD compared with those reporting less than four amenities.

Social support was associated with the mental health outcomes. Scoring one additional point on the social support scale resulted in a decrease in odds of depressive caseness of 3% and anxiety caseness of 2%. There was a decrease in odds of depressive caseness of 10%, anxiety caseness of 8% and PTSD caseness of 6% with each extra point on the family social support subscale. Each additional ‘close person’ reported decreased the odds of depressive caseness by 12% and each additional ‘close friend’ decreased the odds of anxiety caseness by 13%. Higher parental monitoring was associated with lower odds of depressive caseness. High levels of family social support (OR=0.91, 95% CI 0.88–0.96) and higher levels of parental monitoring (OR=0.90, 95% CI 0.82–0.98) were associated with lower odds of suicide attempts.

The best fitting models for depressive and anxiety caseness included adjustments for gender and social support and PTSD adjustments for gender, ethnicity and social support (Tables 3). The social position variables, health behaviours variables and general health variable were not retained in the models as they did not result in any improvement to the models.

**Table 3:** Multivariate analysis: exposure to violence on the Harvard Trauma Questionnaire (HTQ) and depressive, anxiety and post-traumatic stress disorder (PTSD) symptoms on the Short Moods and Feelings Questionnaire, the Self-Rating Anxiety Scale and HTQ

	Depressive symptoms case OR (95% CI)	*P*	Anxiety symptoms case OR (95% CI)	*P*	PTSD symptoms case OR (95% CI)	*P*
Quartile on HTQ adjusting for gender						
1	1	0.016^a^	1	0.011^b^	1	0.10
2	1.76 (0.92–3.35)		2.02 (0.91–4.46)		2.14 (0.38–12.16)	
3	3.17 (2.07–4.87)		2.23 (0.62–8.00)		2.68 (0.56–12.81)	
4	6.55 (3.13–13.67)		5.86 (2.15–15.97)		10.26 (3.01–35.00)	
Quartile on HTQ adjusting for gender, social support						
1	1	<0.001^b^	1	0.008^b^	1^c^	0.084
2	1.80 (1.17–2.77)		2.04 (0.97–4.29)		2.08 (0.39–10.93)	
3	3.02 (2.37–3.85)		2.12 (0.60–7.53)		2.41 (0.53–10.88)	
4	6.23 (4.20–9.23)		5.40 (2.35–12.41)		8.93 (2.93–27.24)	

a Quadratic trend.

b Linear trend.

c Adjusted for gender, ethnicity and social support.

After adjusting for gender, being in the top quartile for violence exposure was associated with greater odds of depressive caseness (OR=6.55, 95% CI 3.13–13.67) and anxiety caseness (OR=5.86, 95% CI 2.15–15.97) compared with those in the bottom quartile. Social support only minimally mitigated the impact of violence exposure on depressive and anxiety symptoms. After adjusting for gender, being in the top quartile of violence exposure was associated with more than ten times the odds of exhibiting PTSD caseness (OR=10.26, 95% CI 3.01–35.00). Adjustment for ethnicity and social support reduced the odds of PTSD caseness to just under nine times those for adolescents in the least exposed category. In the analyses of violence and depressive symptoms adjusted for gender, there was a quadratic trend which was no longer seen after further adjustment. For PTSD, comparing linear with quadratic trends, the quadratic model was a better fit. However, the categorical model with no assumptions was a slightly better fit than the quadratic model.

There was no evidence for an interaction between social support and violence exposure for any of the mental health outcomes.

## Discussion

We found high levels of exposure to violence in this community sample (84.1%). North American (prevalence of community violence: 50–96% girls;^5^ 38% boys^6^) and South African studies of young people^25^ (prevalence of community violence: 94% girls; 90% boys^7^) show a range of prevalence of community violence exposure for adolescents, and our results are at the higher end. We found that exposure to violence was more likely in boys than girls, in those smoking or using alcohol and drugs, in those not living with their father or their father being unemployed, in those living in poor housing and having financial difficulties. The odds of having depressive symptoms, anxiety symptoms and PTSD symptoms were very high in those exposed to the highest quartile of violence exposure and the odds for these disorders increased with increasing degree of exposure to violence.

There may be several explanations. First, young people with existing mental health problems may seek out violent situations, either as witnesses or engaged as perpetrator or victim.^26^ Those with depression who have experienced previous victimisation may not avoid putting themselves in dangerous situations.^27^ There is some limited evidence that young people with emotional disorders especially anxiety and depression, but not conduct disorders with high impulsivity, are more likely generally to avoid violent situations than seek them out.^27^

Second, there may be factors that predict both exposure to violence and independently mental ill health. Factors that predict both violence exposure and mental ill health include socioeconomic status and closely linked to that, socially classified racial group. There is some evidence for socially classified racial group being a common factor with Black students being more at risk for exposure to violence but socioeconomic status does not explain the association of violence and mental ill health, although both violence and mental ill health are related to socioeconomic position in this sample.^13^ Poverty is relevant, especially through increasing the risk of exposure to violence, to polyvictimisation^28^ and revictimisation. High scores on the HTQ in this study are likely to indicate polyvictimisation and multiple rather than single exposure to trauma has been associated with increased risk of PTSD.^29^ Polyvictimisation has been more strongly associated with mental health symptoms in youth from low-income rather than high-income families^12^ which may also be the case in this study. Moreover, community level sexual violence and household violence have been linked to an increased risk of physical and sexual abuse of children so that children living in a community with high levels of violence may increase the risk of exposure to other forms of violence. In a South African study, poverty was found to be a mediator between family AIDS illness and child abuse victimisation relevant to this study population.^30^ Thus poverty might increase the risk of exposure to violence in terms of frequency and duration and limit the social resources that moderate the effects of violence on mental health. Poverty might also increase the risk of victimisation as well as witnessing violence, the former accompanying a greater risk of mental ill health.^31^ Victimisation has been related to a greater risk of emotional disorders than witnessing violence.^32^ Severe victimisation has been related to depression and behavioural problems, and witnessing severe violence has been related to a greater risk of PTSD and delinquency.^33^ Witnessing violence, in turn, has also been related to a greater risk of violence victimisation.

Third, exposure to violence is a causal factor in a wide range of emotional disorders, and given the strength of the associations is the most likely explanation in this study. Among adolescents from the USA, exposure to violence in the community has been longitudinally associated with PTSD and externalising disorders^5,34^ with strong effects for PTSD and externalising disorders and consistent associations between violence and depressive and anxiety disorders and common mental disorders, at ages 18–26 years.^35^ In South Africa, exposure to violence has been linked in cross-sectional studies to PTSD^36^ and depressive symptoms.^7^ Our results are highly consistent with these studies demonstrating strong associations with PTSD and anxiety and depressive symptoms.

Perceived social support, especially from family, has been shown to reduce the risk of PTSD and suicide attempts. Social support had minimal buffering effects in this study, in contrast to many previous studies. This may reflect the special circumstances of this group with high levels of historical and current racism, racial tensions and recurrent violence itself which may undermine possibilities for developing and maintaining useful social support.^2^ Social capital has been found to have little impact on mental health in similar communities exposed to high levels of violence,^35^ and peer support, but not parental warmth or sense of community, had only a small effect on risk of adolescent suicide attempts in a disadvantaged community.^37^ In communities where family relationships are under strain, there is little trust between neighbours and an emphasis on surviving day to day; this may preclude usual social reciprocity and there may be little basis for shared social capital to protect the mental health of adolescents against the effects of violence.

### Limitations

The cross-sectional nature of the study is a limitation and precludes definitive causal interpretations. Our sample was confined to urban areas, but it was representative of Cape Town and covered a wide social spectrum of Cape Town residents; the results may be generalisable to other urban areas with high levels of poverty and social inequalities. A limitation was that both our exposure and health outcomes were measured by self-report questionnaires. However, the measures showed fair internal consistency and retest reliability in a pilot study^15^ but we lacked external corroboration of reports of violence. Our power calculation indicated that we probably lacked sufficient power to test for interactions although our sample size was sufficient for testing our first hypothesis.

### Implications of the study

Burden of disease calculations suggest that interpersonal violence is the second leading cause of healthy years of life lost in South Africa.^37^ Thus, interventions to improve public health in young people could focus predominantly on reducing violence. However, reduction of violence requires interventions at many levels, reducing social inequalities and poverty, reducing unemployment, strengthening families, reducing alcohol misuse, restricting access to firearms coupled with a strong overall political commitment^38^ some of which is underway.^39^ Reduction of poverty may be a critical first step in these interventions. There is also a need for programmatic interventions in vulnerable populations promoting healthy school environments. From the research perspective, there is a need for a better understanding of violence in this specific context in order to design effective interventions, which can be tested using trial methodology.^40^
